# Perspectives of Esophageal Cancer Survivors on Diagnosis, Treatment, and Recovery

**DOI:** 10.3390/cancers13010100

**Published:** 2020-12-31

**Authors:** Annemarie E. Bennett, Linda O’Neill, Deirdre Connolly, Emer Guinan, Lauren Boland, Suzanne Doyle, Jacintha O’Sullivan, John V. Reynolds, Juliette Hussey

**Affiliations:** 1 Discipline of Clinical Medicine, Trinity Centre for Health Sciences, Trinity College, D08W9RT Dublin, Ireland; 2 Discipline of Physiotherapy, Trinity Centre for Health Sciences, Trinity College, D08W9RT Dublin, Ireland; oneilll8@tcd.ie (L.O.); jmhussey@tcd.ie (J.H.); 3 Discipline of Occupational Therapy, Trinity Centre for Health Sciences, Trinity College, D08W9RT Dublin, Ireland; connoldm@tcd.ie (D.C.); laboland@tcd.ie (L.B.); 4 Trinity Centre for Health Sciences, School of Medicine, Trinity College, D08W9RT Dublin, Ireland; guinane1@tcd.ie; 5 School of Biological and Health Sciences, City Campus, Technological University Dublin, D07ADY7 Dublin, Ireland; suzanne.doyle@tudublin.ie; 6 Trinity Translational Medicine Institute, Trinity College, D08W9RT Dublin, Ireland; osullij4@tcd.ie; 7 Discipline of Surgery, Trinity Centre for Health Sciences, Trinity College, D08W9RT Dublin, Ireland; reynoldsjv@stjames.ie

**Keywords:** esophageal cancer, cancer diagnosis, cancer treatment, rehabilitation, patient education, education needs, qualitative

## Abstract

**Simple Summary:**

An esophageal cancer diagnosis signals the start of a difficult period of health-related physical, psychological, and social challenges. To date, relatively few studies have explored the diagnosis, treatment, and recovery experiences of esophageal cancer survivors. Esophageal cancer diagnosis and treatment pose challenges to all aspects of wellbeing, and necessitate an extended period of recovery. As such, supportive education and rehabilitative interventions must encompass a range of strategies to help survivors maintain an adequate quality of life during treatment and recovery. This study aimed to examine patient experiences of esophageal cancer diagnosis, treatment, and recovery, to enable researchers and health professionals to better understand the education and rehabilitative needs of esophageal cancer survivors.

**Abstract:**

Esophageal cancer poses challenges to all domains of wellbeing. This qualitative study aimed to explore the experiences of esophageal cancer diagnosis, treatment, and recovery, with a view to informing the health education needs of this group. Eighteen persons who had undergone an esophagectomy participated in one of four audio-taped focus groups in a specialist hospital for cancer care. Transcriptions were analyzed thematically. Fear and uncertainty underpinned all stages of diagnosis, treatment, and recovery. Participants emphasized: (a) a lack of understanding over what to expect throughout treatment and recovery; (b) the demanding and traumatic period of adjustment required as a result of changes to their physical, psychological, and social functioning; and, (c) that support provided by family, friends, and acquaintances was variable and uninformed, often to the point of being counterproductive to physical and psychosocial recovery. Tailored education is needed to enable patients to prepare for each stage of their cancer journey. Equally, families and wider social networks should receive education that enables them to provide esophageal cancer survivors with appropriate support. Education should be provided at intervals that enable patients, survivors, and support networks to prepare for the physical, emotional, and social challenges experienced during diagnosis, treatment, and recovery.

## 1. Introduction

Despite significant surgical and multimodality advances, esophageal cancer carries a relatively poor prognosis, with a five-year survival rate of approximately 20%. Diagnosis of esophageal cancer often occurs at more advanced stages of the disease [1], and an esophagectomy, alone or in combination with chemotherapy or radiotherapy, is the mainstay of treatment. An esophagectomy is one of the most complex cancer surgical procedures, with an in-hospital mortality rate of up to 5% [2] and a recovery period that may extend beyond one year [3]. Patients commonly incur physical decrements such as muscle loss, food-related difficulties, and fatigue, during and in the aftermath of, treatment. These decrements, in turn, affect emotional wellbeing and health-related quality of life (HRQOL) [4,5,6].

An esophageal cancer diagnosis signals the start of a difficult phase that incurs significant physiological, psychological, and social challenges, to include developing illness coherence, recognizing and accepting a changed self, moving away from self-blame, and regaining agency over day-to-day life [7].

To date, relatively few studies have qualitatively explored the experiences of esophageal cancer survivors. Since diagnosis and treatment pose challenges to all domains of wellbeing [5,7,8], supportive education and rehabilitative interventions must encompass physical, emotional, and social strategies to help survivors attain and maintain an acceptable quality of life.

This study aimed to examine patient experiences of esophageal cancer diagnosis, treatment, and recovery, with a view to informing the content of education and rehabilitative programs for esophageal cancer survivors in a specialist center.

## 2. Materials and Methods

Individuals who had undergone an esophagectomy with curative intent in the previous five years, and who were in remission, were eligible to take part. An invitation to participate in a focus group was issued to 20 esophageal cancer survivors engaged in research activities in the recruiting hospital [9,10]. The written invitation outlining the purpose of the study was issued by a researcher known to potential participants. Those invited had two weeks to consider the invitation.

Sociodemographic data was recorded from participants and their available medical records, and wellbeing was recorded using the Perceived Wellbeing Questionnaire [11], in advance of a focus group starting.

Interviews were facilitated by an occupational therapist experienced in conducting focus groups with persons who received a cancer diagnosis. The facilitators had significant experience in rehabilitation after a cancer diagnosis, both from a clinical and research perspective, and therefore were familiar with the nuances of treatment and recovery. Participants had little or no engagement with the facilitator prior to the focus group taking place. A semi-structured interview guide explored participant experiences of, and needs around, diagnosis, treatment, and recovery [12]. Questions were broad, to encourage discussion, e.g., “What has been the biggest challenge for you since your diagnosis? How would you describe your confidence after your treatment? What are the facilitators/barriers to keeping up good (health) practices after surgery? Have you noticed any changes in your fitness/pain/fatigue since your surgery? How would you describe the role of family and supports throughout diagnosis and treatment, and the time since then?” Interviews took place in the recruiting hospital, and only focus group participants and the facilitator were present. Interviews were audiotaped and lasted an average of 46 min.

Interviews were transcribed verbatim and analyzed thematically [13]. Four authors contributed to the analysis of transcripts. Each author followed a standardized process to analyze transcripts using nVivo 11 (QSR International, Doncaster, Australia), where: codes were systematically generated across the data set; codes were arranged into themes; coded extracts were re-checked to ensure they were congruent with the theme to which they had been assigned; and themes were reviewed to ensure they were clearly defined. A high level of agreement (97%) was apparent when coded transcripts were compared between authors. Any differences in how data were assigned to a theme were discussed and agreement was reached. Given the high rate of recurrence in esophageal cancer survivors, it was deemed inappropriate to return transcripts to participants for comment. The COREQ criteria [14] ensured that the methodology and results were comprehensively presented.

## 3. Results

Eighteen esophageal cancer survivors participated in one of four focus groups, which had two to eight participants each. Socio-demographic data (Table 1) and participant ratings (Table 2) of fatigue, sleep quality, muscle soreness, stress, and mood [11] were recorded. Four themes and 11 sub-themes were constructed from the qualitative data.

### 3.1. Receiving a Diagnosis of Esophageal Cancer

#### 3.1.1. Initial Reaction to a Diagnosis of Esophageal Cancer

Participants discussed their immediate reactions to their diagnosis. They all described the experience in profoundly negative terms, speaking of their mingled dread and anger, as well as an intense sense of loss and upheaval, upon being informed of their diagnosis.

R23: The first time I met [surgeon], he said to me, ‘You’re very sick. You’re going to have to have the operation.’ And I thought, ‘Ah Jesus [irritated tone], what’s this now?’

R08: You are, plain and simple, at your lowest. Your whole world changes.

Some participants acknowledged that they had a limited understanding of cancer prior to their diagnosis. This may have contributed to the fear reported by some participants in the immediate aftermath of their diagnosis, as they learned more about esophageal cancer and its consequences.

R40: I didn’t think about people with cancer, or if they got cancer, or that it shortened their life. But when he took me in and he explained it to me, I thought, ‘This is a whole new ball game here.’

R30: If you go on the Internet, which nobody should do but which everybody does—and I did too—you didn’t see very comforting statistics there, you know? It was… frightening, actually.

Most participants also described the rapid evolution of a profound sense of doubt over the functional capacity of their bodies after diagnosis, and the consequent self-imposed restrictions.

R08: I’d have been afraid to lift that [points to tea tray] after I was told. I’d be afraid, saying, ‘Should I be doing this? Should I be doing that?’ You become very cagey, you know?

#### 3.1.2. Coping with the Reactions of Family and Friends to the Diagnosis

In addition to assimilating their own feelings of anger, loss, fear, and doubt, participants also had to cope with the reactions of family and friends. One participant (R40) highlighted the evasiveness and apparent discomfort he experienced in others, while another (R33) described the disbelief and anger expressed by a sibling in response to her diagnosis.

R40: What I found, in family as well as in friends, is that a lot of people can’t handle the word ‘cancer’. They skirt around it; they treat you as if you are a bloody invalid waiting to go into the box [coffin]!

R33: The whole family was around and all of them were anxious, and when they were told, the first thing my brother said was, ‘She doesn’t meet the profile! How come they picked on her?!’

#### 3.1.3. Strategies for Coping with a Cancer Diagnosis

To help manage the maelstrom of emotion surrounding their diagnosis, most participants agreed on the value of a pragmatic and positive attitude.

R39: That was the danger—to fall into feeling sorry for yourself. But you have to get up, keep going, get on with it, you know?

### 3.2. Navigating Treatment for Esophageal Cancer

#### 3.2.1. Accepting That There Are Tough Times Ahead

Given participants’ understanding of surgery, they expected treatment to be challenging.

R30: You knew they were going to pull your organs around. This operation—it’s as if they take your organs and put them in a bag and shove them up and pour them back into you, so you couldn’t have expected an easy time of it.

However accurate their understanding of the challenges that lay ahead, all participants emphasized the importance of managing their managing their mental health and maintaining a positive attitude during treatment. Several participants highlighted the value of humor as a means of protecting their wellbeing, while others discussed the value of being selective about their company during this difficult time.

R36: I try to be light-hearted—I finds that helps. At home, though, the missus would say to me, ‘You’re very flippant about that.’ And I’d say, ‘No, that’s just the way I deal with it,’ you know?

R41: I wasn’t interested in anybody coming to see me at the time I was going through it. And I made that very clear. I said, ‘I don’t want to entertain anybody that is an extra effort for me.’ It’s a time where you have to mind yourself, and if someone is your friend, they’ll understand that.

#### 3.2.2. Challenging to Engage with Family and Friends during Treatment

About two-thirds of participants acknowledged that while wider family and friends were well-meaning, they could also be overwhelming, since they were inclined to engage with participants at particularly critical and challenging points of their treatment, such as the return home from hospital after surgery.

R40: When I went home, after about a week, I said, ‘Look, I’ll be back in bloody hospital next week, or else I’ll be looking to go back—I can’t cope with this.’ There were people coming in the whole time.

Reflecting some of the challenges in how family and friends responded to their diagnosis and initial treatment, participants discussed how others behaved as treatment progressed. They observed that their social contacts sometimes exhibited excessive positivity and willful oblivion to the physical consequences of treatment, with one participant stating that he found this condescending.

R40: People were coming up to me when I looked dead, saying—’You’re looking great, you’re fantastic.’ And it used to irritate me, because I knew I was looking dead and I felt half-dead!

Another participant highlighted that social contacts can also become desensitized to cancer diagnoses, underpinning this observation with his experience of meeting a colleague.

R36: I met a fellow I worked with on the bus. What was the first thing he said to me? He said, ‘I thought you were dead.’ Well, I won’t tell you what I thought to myself, but it really shocked me when he said that. I got a big setback—it took me a while to get over that.

### 3.3. Early Stages of Recovery after Treatment 

#### 3.3.1. Feeling Disconnected from “Normal” Life

Participants discussed the challenges of the early stages of recovery, with one participant describing her sense of numbness and disconnectedness in the aftermath of treatment, while others described experiencing deep-seated doubts about their ability to resume “normal life”.

R33: I mean, when I came out from the operation, oh my God, for about three months I did nothing. I’d sit in my chair, and then I’d walk into the kitchen and look around the kitchen—I didn’t even recognize it—and walk back to my chair. Those were my days for months.

R08: After everything you’ve been through, your confidence about getting back to normal is shattered.

R23: After the operation, I’d be wondering, ‘Would I be able to do the housework like before, do basic things like keeping the house clean?’

The difficulties posed by routine day-to-day tasks such as eating were also noted.

R39: Learning to eat again had the biggest impact. I had to learn to eat more slowly, because if I didn’t, it would kill you with the pain—horrendous, terrible pain that would take about an hour to clear up.

For most participants, this period of resuming what they described as “normal life”, was disturbed by them feeling hypersensitive to anything that could be perceived as a possible sign of cancer recurrence. Participants recognized this hypersensitivity, observing that they felt it was not reasonable to report all such occurrences to a medical professional.

R11: After this operation, everything changes. You’re saying, ‘There’s pain—what’s that? Should that be there?’ But you can’t be running to the GP every time, you know?

#### 3.3.2. Fatigue Impeded the Return to Daily Activities

All participants identified with, and contributed to, discussion on the impact of fatigue during the early stages of their recovery, with several associating it with their fear of cancer recurrence. For some, this struggle with fatigue negatively altered their perception of themselves, at least temporarily.

R08: When I was told, ‘You’ll feel a bit of fatigue,’ you automatically think, ‘Ah yeah, so I’ll feel a bit tired.’ But fatigue is totally different—you have to explain that it’s a total knackered—all over. And you haven’t done anything, but suddenly you’re knackered and you don’t know why. And it plays on your mind, where you’re saying, ‘What’s gone wrong now that I’m suddenly like this?’

R30: When you’ve those days where you feel physically weak, it’s not necessarily about other people’s view of you—but it’s your view of yourself that’s weaker.

### 3.4. Later Stages of Recovery after Treatment for Esophageal Cancer

#### 3.4.1. Difficulty Establishing a New Routine

As the time from surgery increased, participants described the ongoing difficulty of establishing a routine. This difficulty was partly related to the persistent feeling of doubt over their capacity to resume usual activities.

R33: I remember I couldn’t get back into the swing of things, just not being in the swing of things.

R08: You’re wondering, ‘Is this the right thing to do? Should I be doing this? Should I not be doing this?’ Because you don’t have a clear-cut thing; even though people give you advice, they can’t cover every single thing of every person’s activity, because every person is different.

For example, one participant who was seven months’ post-treatment had recently resumed driving, citing her lack of confidence up to that point in her physical ability to handle driving.

R41: With regard to driving, it’s only starting back with me—whereas during the postoperative period, I just wouldn’t do it because I was afraid of myself from a safety point-of-view and a lack of confidence.

#### 3.4.2. Well-Meaning Family and Friends Inhibited Recovery

Most participants discussed the protective attitude exhibited by their social circle in the later stages of recovery, observing that rather than having a positive effect, such attitudes often suppressed their sense of independence and belief in their ability to recover.

R09: There’s so many people that tell you, ‘Sit down and don’t do anything,’ like, ‘Take it easy, you’re after being through a lot.’ And it’s so easy to buy into that and go down that road of self-pity, to a certain extent, you know?

For example, one participant described how his partner stopped him from gardening.

R40: It’s to do with my other half—she’s over-protective. If I take the sweeping brush out to sweep the garden path, she comes out and takes it off me, and, I do sometimes think, ‘Jaysus, I’m never going to get better.’ Like, I want to do more for myself.

This same participant managed this issue by waiting until his partner left the house, so that he could do his garden work unbeknownst to her.

R40: She’ll say, ‘I’m going into town—do you want to come?’ and I say, ‘No, you go ahead,’ and as soon as she’s gone, the sweeping brush is out and I’m in the garden!

Another participant described how he had to assert himself when his daughter wanted to relieve him of household chores.

R23: My daughter kept saying, ‘I’ll do the housework’ and I kept saying, ‘No—it’s my responsibility. I want to do it.’

This overprotective attitude could be hurtful, particularly when it persisted despite participants making clear that they were motivated to resume the activities of their normal life.

R08: People won’t ask you to do things because they say, ‘We don’t want to ask you.’ And that makes you feel worse because now you feel different to everybody else. You’re desperate to feel normal after it. You know you’ll never be normal again—not the same as you were before—but to the same extent you want to get back as much as you can—to be where you aren’t treated differently to everybody else all of the time.

#### 3.4.3. Identifying a Need for Support and Education

All participants agreed that there was a need for education and support following treatment, and highlighted the limited supports currently available. The potential value of peer support was particularly highlighted, with most participants observing that they had never met another person with esophageal cancer before coming together as a group in the research study. No participant was aware of any support groups for persons with esophageal cancer.

R39: I had never met anyone with esophagus cancer, before this [group].

R01: There should be more support groups. It’s nice to be able to chat to other people—it makes you feel normal—the way you can get past that small talk because somebody knows exactly what you’re talking about.

The limited opportunities within the health system to support and educate patients and their families was also noted.

R33: When you go down to the clinic once a year, you’re just in and out.

The isolation participants experienced with their condition and recovery was evident, with little or no regular or easily accessible formal and informal support available to them or their families and carers. 

## 4. Discussion

This is the first Irish study to explore the perspectives of survivors of esophageal cancer about the period from diagnosis to recovery. It provides insights on the myriad psychosocial challenges experienced by individuals as they cope with being diagnosed with this cancer. The findings emphasize the distress that pervaded all stages of treatment, encompassing participants’ loss of confidence in the capacity of their body to carry out everyday tasks, grief at losing their “normal life”, and apprehension at having to build a “new normal” in the uncertain aftermath of treatment. In addition to their personal struggle with their diagnosis, participants had to cope with the attitudes of family and friends; attitudes which ranged from supportive, to well-meaning-but-undermining, to insensitive. However, for all the difficulties experienced, the importance of a positive outlook as a key coping mechanism was consistently iterated.

Participants described the adverse impact of their diagnosis on their emotional well-being. They described the fear and irritation they felt at having to cope with a cancer diagnosis; something that some had never considered a possibility for themselves. On top of managing their own reaction, there was the emotionally wearing task of coping with outrage, discomfort, and even avoidance, from family and friends when they were informed of the diagnosis. Research consistently reports that individuals with cancer and their families are unified by fear in the face of a life-threatening illness [15,16] and the unknown [17,18]. This fear was sometimes amplified when participants and families attempted to address the “unknown” by conducting Internet searches or speaking with others about the diagnosis. They subsequently became aware of the poor prognosis associated with this cancer [1,19], although not always via health professionals. Since the prognosis is dependent on a range of factors [2,3,4], health professionals should provide tailored information to patients and families on the diagnosis, to equip them with evidence-based information from the outset of their cancer experience. 

To manage the news of diagnosis, participants described the value of coping strategies such as resilient thinking and talking with others, i.e., psychosocial strategies that are recommended by the wider literature [17,20,21,22,23]. These strategies were also important once treatment was underway, but participants highlighted the need to be selective in terms of company kept during this time. For example, one participant described the value of actively limiting contact with specific individuals during treatment, in an effort to conserve her energy and protect her wellbeing. Conversely, another participant described feeling overwhelmed with multiple well-meaning visitors shortly after discharge. Social support independently predicts HRQOL [24,25], making access to informed social support invaluable at a time when HRQOL is threatened [26]. However, a discussion in advance of surgery on managing social support may be helpful, particularly if the discussion includes the primary caregiver, who can be advised on serving as a “gatekeeper” to the patient [17]. When family members act as a buffer between a patient and visitors, this can help patients to better pace their adjustment to any physical and psychosocial challenges that accompany their esophagectomy [6,17].

Participants experienced significant challenges readjusting to life after surgery. They described the trepidation they felt about this adjustment, which seemed to be at least partially related to a fear of cancer recurrence and loss of confidence in the ability of their body to function as expected. Fear of recurrence is common, where symptoms expected as consequences of an esophagectomy are mistakenly interpreted as signs that the disease has returned [16,17,19,27]. For example, fatigue is a common and expected adverse consequence of treatment [12,19], but participants sometimes anxiously interpreted it as a sign that their recovery was not progressing as it should. Even when fatigue was not associated with recurrence, it compounded the decreased confidence some participants had in the ability of their body to manage routine daily activities. The inability to carry out usual activities without a disproportionate sense of tiredness, or even to eat everyday meals without painful consequences, contributed to reduced self-esteem. Physical functioning is not observed to return to baseline in the aftermath of treatment for esophageal cancer [19,28]. Such deficits in function are associated with a poorer sense of self-efficacy, as was evident here, where participants were consistently skeptical of their physical abilities in the aftermath of treatment. To help avert the dissatisfaction that can arise from a discrepancy between expectations and reality, it is important that patients and families are made aware of the physical challenges associated with treatment. However, it is also important that they are made aware that strategies exist to improve, maintain, or attenuate a decline in, physical function; strategies that may, in turn, reduce the emotional and social difficulties related to changes in physical function.

The diagnosis and treatment of, and recovery from, a type of cancer that is unfamiliar to many, may challenge a person’s social network to interact with them in an appropriate manner [6,12]. In this study, the behaviors of others had potentially profound and lasting impacts on participant wellbeing. Just as the individual with cancer must be educated and enabled to cope with a cancer diagnosis and treatment, so too must the individual’s social network. In particular, the way in which a person’s needs change as they move from one phase of cancer to the next, must be recognized [12]. For example, the type of support a person may require during treatment will differ to the support they need as they regain functional capacity after treatment [1,9]. During treatment, intensive physical support may be required. However, support of this intensity may be inappropriate during recovery, since it can disempower individuals to regain their functional capacity, and in turn, impinge upon self-efficacy [12]. Participants described situations in which families did not always recognize the need to lessen their physical support as the time from treatment lengthened; unfortunately, such behaviour tended to impede their recovery and undermined participants’ perceptions of their abilities. Therefore, for social support to be of greatest benefit, it must take the shape of suitably informed individuals who have the capacity to adapt to the changing needs of the patient over time [29,30,31].

Although not specific to individuals with esophageal cancer, self-management support programs implemented by allied health professionals can significantly reduce anxiety, improve self-efficacy, and preserve exercise participation [32,33]. Many of the needs of esophageal cancer survivors can be broadly categorized to reflect those of other cancer survivors [34,35], particularly in terms of fatigue, coping strategies, and a need for adequately informed social support. However, the nuances of esophageal cancer, particularly those related to gastrointestinal health, require particular reassurance and attention, e.g., changes to pace, timing, and volume of meals, urgency of bowel movements, and managing the social implications of these challenges to food intake and digestion. In addition, as a poor prognosis cancer, the comparatively limited number of persons who develop esophageal cancer and survive beyond one year post-surgery, can result in an intensely isolated path to recovery, as opportunities to engage with others in similar situations is limited. Reflecting this, the participants in this study highlighted a lack of consistent and easily accessible professional and peer support and education during their recovery. Further research is needed in this area [36], and given the traumatic experience of esophageal cancer [6,17], research that quantifies the impact of equipping individuals with esophageal cancer, their families, and their social networks, with skills to manage cancer treatment and recovery, is worthy of consideration.

We acknowledge limitations to the study. Given the qualitative design, results are not representative; rather, they provide useful insights that can be difficult to present quantitatively [37]. Length of time from surgery to participating in the study ranged from 8 to 62 months, so recall bias must be considered when interpreting findings. It must also be considered that participants had completed an exercise-based RCT [38] and may represent a more motivated subgroup of individuals recovering from esophageal cancer. 

## 5. Conclusions

This is the first Irish study to explore the perspectives of esophageal cancer survivors on their experience of diagnosis, treatment, and recovery. It highlights the need to develop tailored education and support, not only for those with cancer, but also for families and wider support networks, so that all those affected are equipped and empowered with the skills and strategies needed to handle the physical, emotional, and social challenges likely to be encountered during this challenging time.

## Figures and Tables

**Table 1:** Characteristics of 18 esophageal cancer survivors participating in focus groups.

Study ID	Sex	Age (Years)	Stage at Diagnosis	Neoadjuvant Treatment	Adjuvant Treatment	Type of Esophagectomy	Time Since Surgery(Months)	Employment Status
RESTORE01	F	54	T*is* N0 M0	Nil	Nil	Transhiatal	62	Employed full-time
RESTORE02	M	74	T3 N1 M0	CT + RT (CROSS)	Nil	2-stage	30	Retired
RESTORE04	M	74	T1 N0 M0	Nil	Nil	Transhiatal	23	Retired
RESTORE07	M	81	T1 N0 M0	Nil	Nil	Transhiatal	44	Retired
RESTORE08	M	65	T3 N0 M0	CT + RT (CROSS)	Nil	2-stage	10	Employed part-time
RESTORE09	M	74	T3 N1 M0	CT (MAGIC)	Nil	Not available	35	Retired
RESTORE11	M	61	T1 N0 M0	Nil	Nil	Transhiatal	23	Retired
RESTORE13	F	63	T1 N0 M0	Nil	Nil	3-stage	37	Retired
RESTORE16	M	57	T2 N0 M0	Nil	Nil	2-stage	12	Employed part-time
RESTORE23	M	71	T1 N0 M0	Nil	Nil	Not available	17	Retired
RESTORE26	M	58	T3 N1 M0	CT (MAGIC)	CT	Transhiatal	8	Employed full-time
RESTORE30	M	63	T*is* N0 M0	Nil	Nil	2-stage	10	Employed full-time
RESTORE31	M	74	T3 N1 M0	CT + RT	Nil	2-stage	36	Retired
RESTORE33	F	68	T3 N1 M0	CT + RT (CROSS)	Nil	Transhiatal	42	Retired
RESTORE36	M	63	T1 N0 M0	Nil	Nil	Transhiatal	8	Retired
RESTORE39	M	74	T3 N2 M0	CT + RT (CROSS)	Nil	2-stage	9	Employed part-time
RESTORE40	M	80	T2 N0 M0	Nil	Nil	2-stage	16	Semi-retired
RESTORE41	F	67	T1 N1 M0	CT	CT	3-stage	7	Retired

M = Male; F = Female; CT = Chemotherapy; RT = Radiotherapy; CROSS = ChemoRadiotherapy for Oesophageal cancer followed by Surgery Study; MAGIC = Medical Research Council Adjuvant Gastric Infusional Chemotherapy.

**Table 2:** Responses of 18 esophageal cancer survivors to the Perceived Wellbeing Questionnaire [11].

Study ID	Fatigue	Sleep Quality	Muscle Soreness	Stress Levels	Mood
RESTORE01	Normal	Good	Normal	Normal	Less interested in others/activities
RESTORE02	Normal	Difficulty falling asleep	Normal	Normal	Generally good mood
RESTORE04	Normal	Good	Normal	Normal	Generally good mood
RESTORE07	More tired than normal	Good	Normal	Normal	Generally good mood
RESTORE08	More tired than normal	Good	Feeling great	Relaxed	Very positive mood
RESTORE09	Normal	Good	Normal	Normal	Generally good mood
RESTORE11	Always tired	Restless sleep	Increase in soreness	Feeling stressed	More snappiness at others
RESTORE13	Always tired	Restless sleep	Very sore	Feeling stressed	Less interested in others/activities
RESTORE16	More tired than normal	Good	Increase in soreness	Feeling stressed	Less interested in others/activities
RESTORE23	Normal	Very restful	Feeling good	Feeling stressed	Generally good mood
RESTORE26	Normal	Good	Normal	Relaxed	Generally good mood
RESTORE30	Normal	Good	Normal	Normal	More snappiness at others
RESTORE31	Always tired	Good	Feeling good	Normal	Less interested in others/activities
RESTORE33	Missing	Missing	Missing	Missing	Missing
RESTORE36	More tired than normal	Good	Normal	Feeling stressed	Less interested in others/activities
RESTORE39	Normal	Restless sleep	Normal	Normal	Generally good mood
RESTORE40	Normal	Good	Normal	Very relaxed	Generally good mood
RESTORE41	More tired than normal	Restless sleep	Increase in soreness	Normal	Less interested in others/activities

## Data Availability

The anonymized datasets used and/or analyzed during the current study are available from the corresponding author on reasonable request.
